# Cognitive and structural brain changes in ofatumumab-treated multiple sclerosis: A longitudinal study

**DOI:** 10.1016/j.neurot.2026.e00922

**Published:** 2026-06-03

**Authors:** Aurora Zanghì, Paola Sofia Di Filippo, Carlo Robusto, Maria Claudia Moretti, Carlo Avolio, Emanuele D’Amico

**Affiliations:** BRAND (Breakthrough Research in Autoimmune and Neurodegenerative Diseases) Research Center, Department of Medical and Surgical Sciences, University of Foggia, Italy

**Keywords:** Multiple sclerosis, Ofatumumab, Neurodegeneration, Cognitive impairment, Brain atrophy

## Abstract

Anti-CD20 therapies control inflammatory activity in multiple sclerosis (MS), but their effects on neurodegeneration and cognition in real-world settings remain uncertain. We conducted a longitudinal observational study of relapsing MS (RMS) patients treated with ofatumumab. Cognitive performance was assessed using the Brief International Cognitive Assessment for Multiple Sclerosis (BICAMS), with processing speed as the primary outcome, and Magnetic Resonance Imaging (MRI) measures included thalamic volume, deep gray matter (DGM) volume, and cortical thickness (CTh). Longitudinal changes and structure–cognition associations were analyzed using linear mixed-effects models. Eighty-five RMS patients treated with ofatumumab (mean age 37.9 ± 9.9 years; 69.4% female; 222 MRI scans) were included. Brain atrophy persisted but attenuated beyond 12 months: whole-brain volume −0.15% to −0.09%; DGM −1.01% to −0.36%; thalamus −0.94% to −0.77%; CTh –1.67% to –0.62% (all p for slope changes >0.05). Cognitive performance increased (Symbol Digit Modalities Test, SDMT +3.79 points/year, 95% CI 1.35 to 6.23; *p*= 0.003), with increases in verbal and visuospatial memory (Brief Visuospatial Memory Test–Revised and California Verbal Learning Test–Second Edition, *p*< 0.05). No global longitudinal structure–cognition association was observed. In subgroups, thalamic atrophy was associated with SDMT decline in patients with Expanded Disability Status Scale ≥3 (*β*= −54.31; *p*= 0.013), and DGM atrophy with cognitive worsening in patients with baseline SDMT z-score ≤ −1 (*β*= −55.91; *p*= 0.012). In ofatumumab-treated relapsing MS, neurodegeneration persists but slows over time while cognition may be preserved, and structural–functional coupling emerges only in vulnerable patients.

## Introduction

Multiple sclerosis (MS) is a chronic inflammatory disease of the central nervous system (CNS) in which neurodegeneration contributes to long-term disability [1]. The introduction of anti-CD20 monoclonal antibodies has substantially improved the control of inflammatory disease activity in relapsing MS (RMS), leading to marked reductions in relapse rates and magnetic resonance imaging (MRI) inflammatory activity [2,3]. However, long-term disability in MS is only partially explained by focal inflammation, and neurodegenerative processes may continue to contribute to disease progression despite effective immunotherapy [[4], [5], [6]].

Structural MRI provides a sensitive means of quantifying neurodegeneration through the assessment of regional brain tissue loss. Deep gray matter (DGM) structures, particularly the thalamus, as well as cortical measures, have consistently shown strong associations with physical disability and cognitive dysfunction [7]. Cognitive impairment represents a major determinant of functional limitation and quality of life in MS and may be present even in patients with low levels of neurological disability [8].

Despite the effectiveness of anti-CD20 therapies in suppressing inflammatory activity, the longitudinal relationship between structural brain changes and cognitive function under these treatments remains incompletely understood, particularly in real-world clinical practice. Structural damage may continue to accumulate under therapy, while its functional consequences may not be immediately apparent. Clarifying whether, and under which conditions, longitudinal brain tissue loss translates into cognitive change is therefore a key unmet need.

Ofatumumab is a fully human anti-CD20 monoclonal antibody administered subcutaneously and is increasingly used as a high-efficacy therapy early in the disease course [9,10]. Its widespread use in routine clinical practice provides an opportunity to investigate longitudinal structural and cognitive outcomes in a heterogeneous patient population beyond the constraints of randomized clinical trials [11,12].

In addition to imaging and clinical measures, fluid biomarkers may offer complementary information on ongoing neuroaxonal injury. Neurofilament light chain (NfL) has emerged as a marker of neuronal damage and inflammatory activity in MS; however, its ability to predict longer-term structural and cognitive outcomes under effective immunotherapy remains uncertain [13].

In this longitudinal real-world study, we aimed to: 1) characterize longitudinal changes in regional brain structure in MS patients treated with ofatumumab; 2) describe longitudinal cognitive trajectories under treatment; 3) assess the association between structural brain changes and cognitive evolution over time; and 4) explore baseline serum and cerebrospinal NfL (sNfL and cNfL) as a potential predictor of subsequent structural and cognitive outcomes in treatment-naïve patients.

## Methods

### Standard protocol approvals, registrations, and patient consents

This study was approved by the Bari Ethics Committee (reference: 7908/2025). Informed written consent from participants was obtained from all participants. Data were extracted on December 31st, 2025 from electronic medical records.

### Study setting and design

A retrospective cohort study in RMS patients treated with ofatumumab conducted at the MS center of Foggia, Italy. Clinical, neuropsychological and radiological data were acquired prospectively.

### Participants and inclusion criteria

Consecutive adult patients (≥18 years) with a diagnosis of RMS according to the 2017 McDonald criteria [14] who initiated treatment with ofatumumab between January 2022 and January 2024 were screened for eligibility at the MS Center of Foggia.

Patients were included if they had at least one follow-up MRI examination performed within 12 months after treatment initiation, allowing a ±3-month window. Patients with major psychiatric disorders, or other neurological conditions potentially affecting cognitive performance were excluded.

### Data collection

Treatment initiation with ofatumumab was determined as part of routine clinical care by the treating neurologist and eligibility according to regulatory indications. The study did not interfere with clinical management.

In detail, treatment with ofatumumab consisted of an initial loading phase with 20 mg administered subcutaneously at weeks 0, 1, and 2, followed by maintenance dosing of 20 mg subcutaneously once monthly beginning at week 4.

Data were recorded retrospectively (including data until one year before baseline) and prospectively (until the last available visit of follow-up). Baseline demographic and clinical characteristics, including comorbidities, body mass index (BMI), disease duration, relapse history, and prior exposure to disease-modifying therapies (DMTs), were extracted from electronic medical records. Clinical records were reviewed to identify comorbidities and concomitant medications potentially affecting cognitive performance. Disease duration was defined as the time interval between MS onset and the study baseline.

Disability was assessed with the Expanded Disability Status Scale (EDSS) performed by a Neurostatus-certified MS specialist, at baseline and during follow-up visits. Number of relapses during the 12 months before treatment initiation with ofatumumab and total number of relapses during ofatumumab treatment were recorded. Relapses had a standardized definition [15] and were defined as the onset of new neurological symptom(s) or the exacerbation of existing symptom(s) lasting for at least 24 h in the absence of concurrent illness or fever, occurring at least 30 days after a previous relapse. Relapses are usually categorized into seven phenotypes based on the presenting symptoms and signs: pyramidal, sensory, bowel/bladder, cerebellar, brainstem, visual and cognitive as previously defined [15].

### Cognitive assessment

Patients underwent cognitive assessment through the Brief International Cognitive Assessment for Multiple Sclerosis (BICAMS), administered by an experienced neuropsychologist. The battery includes the Symbol Digit Modalities Test (SDMT), the California Verbal Learning Test–Second Edition (CVLT-II), and the Brief Visuospatial Memory Test–Revised (BVMT-R).

Assessments were performed at baseline and at 12-month intervals during follow-up, allowing a ±3 month window. Raw scores and z-scores for each test were collected for all available assessments. Z-scores were calculated using published normative data from the Italian validation of the BICAMS battery, applying demographic corrections for age, sex, and education [16].

### MRI acquisition

Imaging was performed on either a 3-T MRI scanner (Siemens, Magnetom Skyra) or a 1.5-T MRI scanner (Philips, Achieva dStream). Both acquisition protocols included a 3-dimensional (3D) T1-weighted sequence and a 3-D, 1-mm isotropic fluid attenuated inversion recovery (FLAIR) sequence. The details of T1-weighted sequences acquisition protocols are described in Supplementary Table 1.

### MRI analysis

Baseline MRI was defined as the scan acquired within a −3 to +1 month window relative to treatment initiation. Follow-up MRI was defined as the scan acquired at 12 months after treatment initiation, with a ±3-month allowable window. All available longitudinal MRI examinations beyond the first year of therapy meeting these criteria were included in the analysis.

MS lesions were segmented by the lesion prediction algorithm (LPA) implemented in the LST toolbox version 3.0.0 for SPM on the 3D-FLAIR sequences, allowing quantification of the T2-hyperintense lesion volume (T2LV) [17]. Then, the resulting probability lesion maps were used to perform lesion filling of 3D-T1 images, to reduce potential variation in volumetric analysis due to MS lesions.

Volumetric analysis was performed on lesion-filled 3D-T1-weighted images with the longitudinal pipeline of FreeSurfer (version 8.0.0) [18]. Such processing stream allows for the creation of an unbiased within-subject template; then, common information from such template is used to initialize the processing for each time point [19]. This approach is associated with an improvement in sensitivity and statistical power in detecting subtle longitudinal changes [19].

As regions of interest (ROIs), we considered total brain volume (TBV), cerebral cortex, total GM, total white matter (WM), cerebellum, thalamus and estimated total intracranial volume (eTIV).

FreeSurfer’s output provides TBV as a direct estimation, whereas all other volumetric measures are computed separately for the left and right hemispheres [18]. Hence, for symmetrical structures, left and right volumes directly segmented by FreeSurfer were summed and the DGM volume was calculated as the sum of the thalamus, caudate, putamen, pallidum, nucleus accumbens, hippocampus and amygdala, as previously described [20].

Mean cortical thickness (CTh) was quantified for the whole cortex, as well as for each lobar and regional cortical area (according to the Desikan-Killiany atlas), as the average of the thickness obtained in the two hemispheres.

### Laboratory procedures and NfL assessment

sNfL and cNfLs concentrations were measured using the ELLA technique with a Simple Plex Human NF-L Cartridge®, following a 1:2 dilution, with all samples processed in duplicate and demonstrating coefficients of variation <10% [21].

### Statistical analysis

Baseline demographic, clinical, cognitive, MRI, and biomarker characteristics were summarized using mean and standard deviation or median and quartiles (first and third) as appropriate, for continuous variables, and counts with percentages for categorical variables, respectively. The normality and asymmetry of continuous variables’distribution were assessed by skewness index and Q–Q plot.

All analyses followed STROBE recommendations for observational studies.

#### Longitudinal modeling framework

Longitudinal analyses were performed using linear mixed-effects models (LME) to account for repeated measurements within subjects and variable follow-up intervals. Time since ofatumumab initiation was modeled as a continuous variable (in years). All models included subject-specific random intercepts to account for within-subject correlation; an additional random intercept for MRI acquisition protocol was included to account for between-scanner variability.

To address collinearity between baseline age and disease duration, a conditioned disease duration covariate (CDA) was derived by regressing disease duration on age at baseline and including the resulting residuals as a covariate in all models.

#### Longitudinal structural MRI analyses

Brain volume measures were log-transformed prior to analysis, allowing model coefficients to be interpreted as relative annual percentage changes (APC). To evaluate whether rates of structural change differed between early and later treatment phases, a piecewise linear term with a knot at 12 months from treatment initiation was included. Models were adjusted for sex, CDA, and log-transformed eTIV to account for between-subject differences in head size. Annual percentage change estimates for early (≤12 months) and later (>12 months) phases were obtained by back-transforming coefficients from the log scale.

#### Longitudinal cognitive analyses

Cognitive outcomes were analyzed using LME applied to BICAMS scores, with SDMT prespecified as the primary endpoint. SDMT was prespecified as the primary cognitive outcome, consistent with its widespread use as a sensitive cognitive endpoint in multiple sclerosis clinical trials [22]. Primary analyses were conducted on raw scores to preserve within-subject variability over time. Time since treatment initiation was modeled as a continuous variable, and the same longitudinal modeling strategy described for structural analyses was applied. For consistency with structural models, a piecewise time term at 12 months (change in slope) was included in cognitive LME models; this term represents a change in the longitudinal trajectory rather than a categorical pre/post treatment effect Models were adjusted for sex, education level, and CDA, with subject-specific random intercepts to account for repeated measurements.

#### Structure–cognition relationships

Associations between MRI-derived structural measures and cognitive performance were examined using within–between (Mundlak) LME models. For each MRI measure, the subject-specific mean across visits (between-subject effect) and the deviation from that mean at each visit (within-subject effect) were entered simultaneously into the model, allowing separation of cross-sectional associations from true longitudinal coupling. Models included time since treatment initiation, sex, education, CDA, and eTIV for volumetric measures, with random intercepts for subjects.

To account for baseline disability, these models were repeated with additional adjustment for baseline EDSS.

#### Exploratory subgroup analyses (Δ–Δ ANCOVA)

Given limited sample sizes in clinically and cognitively vulnerable subgroups, longitudinal coupling was further explored using 12-month change–change analyses. For each subject, changes in cognitive performance and MRI measures were calculated as the difference between baseline and the visit closest to 12 months. Separate analysis of covariance (ANCOVA) models were fitted with 12-month change in cognitive performance as the dependent variable and 12-month change in MRI measures as the independent variable.

Analyses were performed in predefined subgroups defined by baseline disability (EDSS ≥3) and baseline cognitive impairment (SDMT z-score ≤ −1). Models were adjusted for sex, education, CDA, and log-transformed eTIV for volumetric measures. These analyses were prespecified as exploratory and hypothesis-generating.

#### Serum neurofilament light chain analyses

In a subset of treatment-naïve patients with available baseline sNfL and cNfL measurements (Log-transformed), two ANCOVA models were used to assess whether baseline sNfL and cNfL predicted subsequent structural or cognitive outcomes. Models were adjusted for sex and CDA, and for eTIV. Given the skewed distribution of sNfL and cNfL and the limited sample size, sensitivity analyses were performed using winsorization at the 95th percentile and outlier-robust specifications. Consistency of results across approaches was used to assess robustness.

All statistical tests were two-sided, with statistical significance set at *p*< 0.05. Data were complete and no imputation for missing data was done. Given the exploratory nature of subgroup and biomarker analyses, no formal correction for multiple comparisons was applied. Effect estimates and 95% confidence intervals are reported throughout. All analyses were conducted using R (version 4.5.1).

### Data availability

Any data not presented in the article will be made available on request to any senior (tenured) investigator.

## Results

**Supplementary Figure 1:**
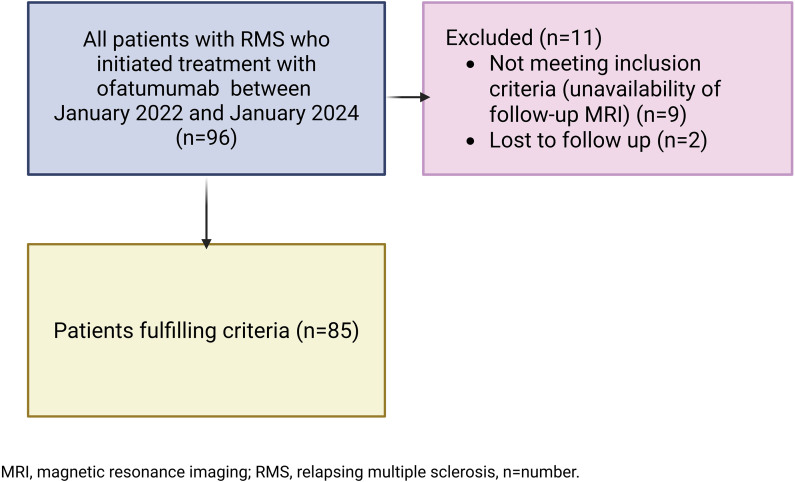
**Flow chart of the study.** MRI, magnetic resonance imaging; RMS, relapsing multiple sclerosis; n = number.

Baseline characteristics of the study cohort are shown in Table 1. Eighty-five patients with RMS initiating ofatumumab were included in the study. The patient selection process and retention during follow-up are illustrated in Supplementary Fig. 1. Mean age at treatment start was 37.9 ± 9.9 years, and 59 patients (69.4%) were female. Comorbidities were present in 35 patients (41.2%), and mean BMI was 25.3 ± 4.9 kg/m^2^. Median disease duration at baseline was 4.5 years (IQR, 0.5–14.7). Forty-five patients (52.9%) were treatment-naïve. In the 12 months preceding enrollment, patients experienced a median of one relapse (IQR, 1–2). Median EDSS at treatment initiation was 2.5 (IQR, 2.0–3.5). Mean duration of ofatumumab exposure during follow-up was 19.7 ± 9.3 months.

**Table 1:** Baseline characteristics of the study cohort.

Variable^a^	Whole cohort (*n*= 85)
Age at start of ofatumumab (years)	37.9 ± 9.9
**Sex**, n (%)	
Male	26 (30.6)
Female	59 (69.4)
**Comorbidities**, n (%)	
Yes	35 (41.2)
No	50 (58.8)
**BMI** (kg/m^2^)	25.3 ± 4.9
**Disease duration** (years)	4.5 (0.5–14.7)
**Naïve to DMTs**, n (%)	45 (52.9)
**N. of prior DMTs**, median (Q1-Q3)	0 (0–2)
**N. of relapses within 12 months before enrollment**, median (Q1-Q3)	1 (1–2)
**EDSS at the time of ofatumumab start**, median (Q1-Q3)	2.5 (2.0–3.5)
**Time on ofatumumab (months)**	19.7 ± 9.3
**SDMT z-score**	−0.65 ± 0.99
**BVMT-R z-score**	−0.72 ± 0.99
**CVLT-II z-score**	−0.52 ± 0.80

BMI, body mass index; BVMT-R, Brief Visuospatial Memory Test–Revised; CVLT-II, California Verbal Learning Test–Second Edition; DMTs, disease-modifying treatments; EDSS, Expanded Disability Status Scale; MRI, magnetic resonance imaging; n, number; N. number; Q1–Q3, quartiles; SD, standard deviation; SDMT, Symbol Digit Modalities Test.

a Data are expressed as median and standard deviation when otherwise specified.

### Baseline MRI findings

At treatment initiation, baseline MRI showed a median T2 lesions volume of 5421.3 mm^3^ (IQR, 3571.4–12,929.5). Median TBV was 1,031,193.0 mm^3^ (IQR, 953,580.5–1,115,079.0), with a mean cortical thickness of 2.5 ± 0.2 mm. Median thalamic volume was 13,130.8 mm^3^ (IQR, 11,711.6–14,500.7), and median DGM volume was 46,229.7 mm^3^ (IQR, 41,812.2–50,201.2) (Table 2).

**Table 2:** Baseline brain MRI volumetric measures and T2 lesions volume.

Variable^a^ (mm^3^)	Whole cohort (*n*= 85)
T2LV	5421.3 (3571.4–12,929.5)
TBV	1,031,193.0 (953,580.5–1,115,079.0)
Cerebral cortex	445,758.9 (412,527.4–499,145.0)
Total GM	611,784.4 (567,944.7–670,993.5)
Total WM	417,631.0 (370,505.0–459,342.0)
Deep GM	46,229.7 (41,812.2–50,201.2)
Cerebellum	139,718.7 (127,447.0–147,749.2)
Thalamus	13,130.8 (11,711.6–14,500.7)

GM, gray matter; MRI, magnetic resonance imaging; TBV, total brain volume; T2LV, T2-lesion volume; WM, white matter.

a Expressed as median and Q1-Q3.

### Baseline cognitive performance

Processing speed assessed by the SDMT showed a baseline mean z-score of −0.65 ± 0.99. Visuospatial memory performance on the BVMT-R was −0.72 ± 0.99, and verbal learning assessed by the CVLT-II was −0.52 ± 0.80 (Table 1).

### Longitudinal MRI changes

**Supplementary Figure 2:**
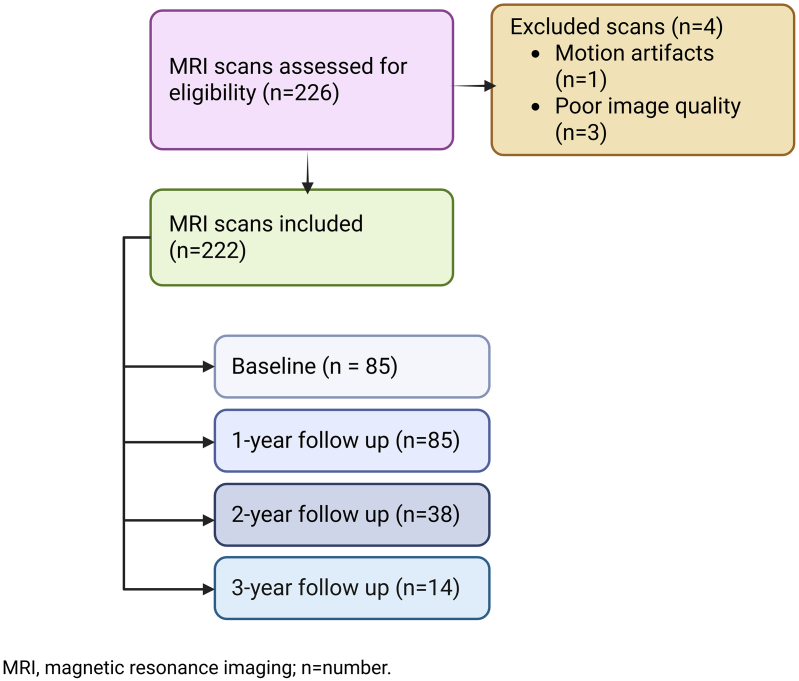
**Flow chart of MRI scans quality control and inclusion in the longitudinal analysis.** MRI, magnetic resonance imaging.

A total of 222 MRI examinations with adequate image quality were included in the longitudinal analyses (Supplementary Fig. 2). Whole-brain volume declined during the first year of treatment, with an estimated APC of −0.15%/year, followed by a numerically lower rate of decline beyond 12 months of treatment (APC −0.09%/year) (Table 3).

**Table 3:** Longitudinal rates of brain volume change during ofatumumab treatment.

ROI	APC ≤12 months (%/year)	APC >12 months (%/year)	Difference in APC	*p*-value (slope change)^a^
**Total brain volume**	−0.15%	−0.09%	+0.06%	0.95
**Cerebral cortex**	−1.10%	−0.36%	+0.74%	0.67
**Total WM**	+0.93%	+0.12%	−0.81%	0.69
**Total GM**	−1.45%	−0.27%	+1.18%	0.39
**DGM**	−1.01%	−0.36%	+0.65%	0.23
**Cerebellum**	−2.22%	−0.50%	+1.72%	0.11
**Thalamus**	−0.94%	−0.77%	+0.17%	0.89

APC: annual percentage change derived from log-transformed mixed-effects models; DGM, deep gray matter; GM, gray matter; WM, white matter; ROI, region of interest.

a Adjusted for sex, conditional disease duration covariate, and estimated total intracranial volume; random intercepts for subject and MRI protocol.

GM compartments showed clearer attenuation patterns over time. DGM volume exhibited a reduction in the rate of decline from −1.01%/year during the first year to −0.36%/year beyond 12 months (*p* for slope change = 0.23). Cortical volume declined at a rate of −1.10%/year during the first year and −0.36%/year thereafter (*p* for slope change = 0.67; Table 3), while total GM volume decreased from −1.45%/year to −0.27%/year over the same periods (*p* for slope change = 0.39; Table 3). Cerebellar volume showed a reduction in the rate of volume loss from −2.22%/year during the first year to −0.50%/year beyond 12 months (*p* for slope change = 0.11; Table 3).

Thalamic volume declined during the first year with an APC of −0.94%/year and showed a lower rate of atrophy beyond 12 months (APC −0.77%/year; *p* for slope change = 0.89) (Table 3).

**Fig. 1:**
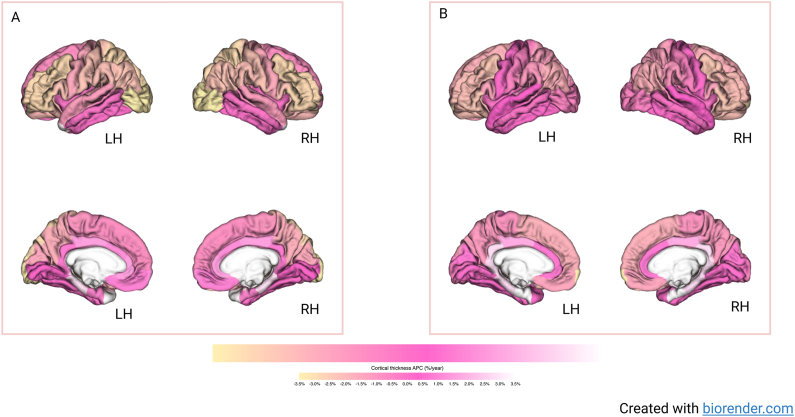
**Lateral and medial views of cortical surface maps illustrating annual percentage change in cortical thicknesses, during the first 12 months of treatment (panel A) and after 12 months (panel B).** LH, left hemisphere; RH, right hemisphere; APC, annual percentual change.

CTh declined during the first year (APC −1.67%/year) and exhibited a slower rate of change thereafter (APC −0.62%/year; *p* for slope change = 0.47) (Supplementary Table 2; Fig. 1). Regional analyses showed attenuated cortical atrophy rates beyond 12 months across all lobes (Fig. 1).

### Longitudinal cognitive trajectories

**Fig. 2:**
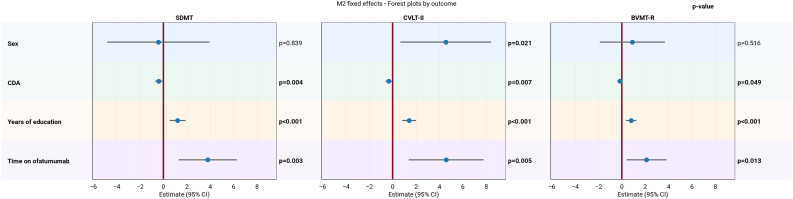
**Forest plots of longitudinal cognitive analysis model.** Results are displayed for three outcomes: SDMT, CVLT-II, and BVMT-R raw scores. Points represent beta coefficients (estimated effect sizes) and horizontal lines indicate 95% confidence intervals. *P*-values are shown in the right-hand columns, with statistically significant *p*-values (*p*< 0.05) highlighted in bold. BVMT-R, Brief Visuospatial Memory Test–Revised; CI, confidence interval; CVLT-II, California Verbal Learning Test–Second Edition; SDMT, Symbol Digit Modalities Test.

Processing speed increased over time, with SDMT increasing by 3.79 points per year (95% CI, 1.35 to 6.23; *p*= 0.003). Improvements were also observed for verbal and visuospatial memory outcomes (all *p*< 0.05; Supplementary Table 3). Education level showed a consistent positive association with cognitive performance across all tests (all *p*< 0.001) (Fig. 2).

### Structure–cognition associations

In within–between LME, SDMT improved over time independently of MRI measures (Table 4). Within-subject changes in thalamic volume were not associated with concurrent changes in SDMT (*β*= −8.54, 95% CI - 25.49 to 8.42; *p*= 0.32). By contrast, between-subject effects were significant, with higher mean thalamic volume associated with better SDMT performance (*β*= 36.56, 95% CI 21.84 to 51.29; *p*< 0.001). Similar significant between-subject associations were observed for deep gray matter volume (*p*< 0.001), whereas between-subject differences in CTh were not significantly associated with SDMT (*p*= 0.153). Results were unchanged after adjustment for baseline EDSS (Supplementary Table 4).

**Table 4:** Association between SDMT raw score and longitudinal MRI changes in cortical thickness, thalamic volume, and deep gray matter volume.

Predictor	Beta	SE	95% CI (lower, upper)	p-value

**CTh changes**				
Intercept	9.504	17.114	-24.559, 43.567	0.58
CDA	-0.382	0.134	-0.649, -0.116	**0.005**
Sex^a^	-0.788	2.160	-5.088, 3.513	0.716
Education, years	1.100	0.320	0.462, 1.738	**<0.001**
Time on ofatumumab treatment, months	3.891	0.560	2.778, 5.004	**<0.001**
Within-subject changes in CTh^b^	-0.595	2.894	-6.351, 5.162	0.838
Between-subjects changes in CTh^c^	10.396	7.200	-3.933, 24.725	0.153
**ThalVol changes**				
Intercept	-121.496	135.392	-389.148, 146.155	0.371
CDA	-0.192	0.123	-0.438, 0.053	0.122
Sex^a^	1.751	2.327	-2.870, 6.371	0.454
Education, years	1.022	0.279	0.466, 1.577	**<0.001**
Time on ofatumumab treatment, months	3.818	0.534	2.757, 4.878	**<0.001**
eTIV	-13.393	10.592	-35.027, 8.240	0.223
Within-subject changes in ThalVol^b^	-8.538	8.526	-25.496, 8.420	0.320
Between-subjects changes in ThalVol^c^	36.563	7.418	21.836, 51.290	**<0.001**
**DGMVol changes**				
Intercept	-206.199	145.592	-494.609, 82.212	0.159
CDA	-0.189	0.124	-0.436, 0.057	0.130
Sex^a^	1.295	2.398	-3.469, 6.058	0.591
Education, years	1.051	0.280	0.494, 1.608	**<0.001**
Time on ofatumumab treatment, months	3.887	0.563	2.767, 5.006	**<0.001**
eTIV	-19.538	12.940	-45.118, 6.042	0.133
Within-subject changes in DGMVol^b^	-3.229	17.016	-36.962, 30.505	0.850
Between-subjects changes in DGMVol^c^	48.328	10.313	27.858, 68.798	**<0.001**

CDA, Conditional Disease Duration covariate; CI, Confidence interval; CTh, Cortical thickness; DGMVol, Deep gray matter volume; eTIV, Estimated total intracranial volume; SDMT, Symbol Digit Modalities Test; SE: Standard error; ThalVol, Thalamic volume.

a Male as a reference.

b Computed as each observation’s deviation from the subject-specific mean.

c Computed as the subject-specific mean across available observations.

### Structure–cognition associations in more disabled subgroups

**Fig. 3:**
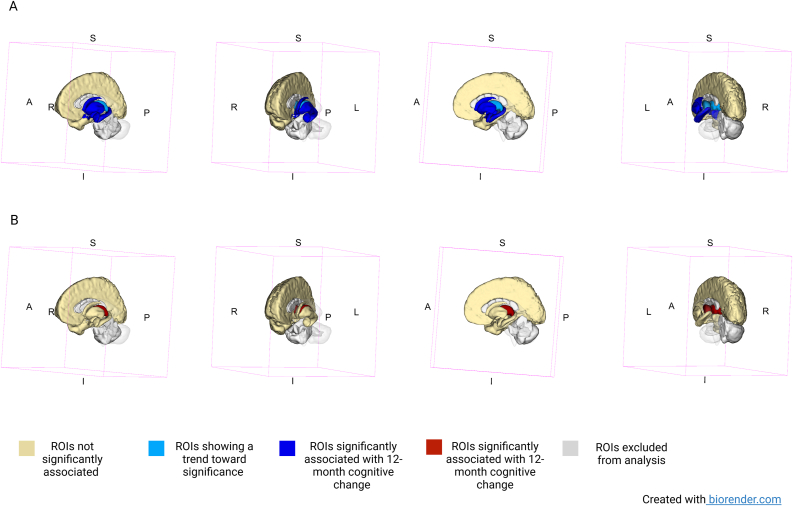
**Associations between 12-month brain volume loss and cognitive decline by clinical subgroup. A. Patients with SDMT z-score ≤ −1. B. Patients with EDSS ≥ 3.** ROIs showing significant correlations between changes in brain volume and SDMT performance are depicted in three-dimensional renderings for each subgroup Significant ROIs are shown in red (Panel A) and blue (Panel B), trend-level associations in light blue (Panel B), and non-significant or excluded ROIs in light yellow and gray, respectively, in both panels. A, anterior; EDSS, Expanded Disability Status Scale; I, inferior; L, left; P, posterior; R, right; ROIs, regions of interest; S, superior; SDMT, Symbol Digit Modalities Test.

In patients with baseline EDSS ≥3, greater 12-month thalamic volume loss was significantly associated with a larger decline in SDMT (*β*= −54.31, 95% CI -95.59 to -13.03; *p*= 0.013) (Fig. 3). In patients with baseline SDMT z-score ≤ −1, 12-month changes in deep GM volume were significantly associated with 12-month SDMT change (*β*= −55.91, 95% CI −98.25 to −13.58; *p*= 0.012), whereas the association with thalamic volume was borderline and did not reach statistical significance (Fig. 3).

### Neurofilament light chain analyses

In ANCOVA models adjusted for baseline values, neither cNfL nor sNfL was significantly associated with 12-month changes in cognitive or MRI outcomes (all *p*> 0.05; Supplementary Table 5A and B). Results were consistent across robustness analyses, including exclusion of extreme and influential observations and winsorization at the 95th percentile (Supplementary Table 6A and B).

## Discussion

This monocentric, longitudinal real-world study provides comprehensive insights into structural, cognitive, and biomarker dynamics in patients with RMS treated with ofatumumab over approximately 19 months. The findings reveal a complex interplay between effective peripheral B-cell depletion, ongoing neurodegeneration, and preserved cognition, highlighting asynchronous and multifaceted disease processes under high-efficacy therapy.

Ofatumumab has been shown to reduce brain volume loss compared with placebo (ratio of means = 0.58; 95% CI, 0.40 to 0.83) and to effectively suppress MRI lesion activity in pivotal trials [23,24]. Despite this potent anti-inflammatory effect, we observed ongoing neurodegeneration affecting deep gray matter and cortical regions. The rate of structural decline did not accelerate beyond 12 months, suggesting partial stabilization. This pattern is consistent with preclinical data showing that anti-CD20 therapy delays GM atrophy and mitigates microglial proliferation, though it does not fully halt neurodegenerative processes [25]. The persistence of atrophy despite effective inflammatory control supports the concept of compartmentalized inflammation, increasingly recognized as a driver of disability in MS that remains partially independent of peripheral immune activity [26].

Recent work on smouldering MS by Scalfari et al. [27] introduced the concept of smouldering-associated worsening (SAW), encompassing subtle physical and cognitive decline caused by CNS-compartmentalized pathology. The ongoing structural loss observed in our cohort in the absence of relapses or new lesions aligns with this SAW framework, suggesting persistent smouldering activity under B-cell depletion.

Despite measurable atrophy, cognitive performance—particularly processing speed—remained stable or slightly improved.

This observation may reflects the influence of functional reserve, enabling maintenance of performance despite progressive structural damage. Patients with preserved cognition and mild disability at baseline likely benefited from compensatory network reorganization, consistent with prior functional imaging studies demonstrating adaptive recruitment of alternative circuits in early MS [28].

At the between-subject level, higher deep GM volume, particularly thalamic integrity, was associated with better cognitive outcomes, as expected. However, within-subject longitudinal associations between structural and cognitive change were observed only among individuals with reduced baseline reserve, supporting a threshold-dependent model in which compensation fails once critical structural loss is reached. This observation aligns with the concepts of progression independent of relapse activity (PIRA) and progression independent of relapse and MRI activity (PIRMA) described by Ciccarelli et al. [29] emphasizing that disability accrual may continue through cortical pathology, slowly expanding lesions, and gray matter atrophy, even in the absence of acute inflammatory activity [30].

In the treatment-naïve subset, baseline sNfL nor cNfLs did not predict subsequent structural or cognitive outcomes. Although NfL is a robust marker of axonal injury, its prognostic value appears limited under potent anti-inflammatory therapy. In this setting, baseline NfL likely reflects pre-treatment inflammatory activity rather than ongoing compartmentalized neurodegeneration. These findings align with emerging evidence that NfL dynamics are temporally complex, with some phenotypes showing gray matter atrophy preceding biomarker elevation (“late-NfL”) [31].

Thus, serial rather than static measurements may better capture subclinical neurodegeneration under sustained B-cell depletion.

Together, these results are consistent with an asynchronous disease model in MS, in which neurodegeneration, inflammation, and clinical disability may evolve on distinct temporal trajectories [[32], [33], [34]].

The coexistence of inflammatory quiescence, progressive structural loss, and preserved function may reflect temporal dissociation across disease domains [35]. This pattern is consistent with both the smouldering MS and PIRA frameworks, emphasizing that relapse-based monitoring underestimates ongoing pathology [27,30].

Our findings highlight the need for multimodal monitoring beyond relapse rates and conventional MRI activity, integrating volumetric imaging, cognitive testing, and fluid biomarkers to detect silent progression. Reserve-stratified management may be warranted, as patients with lower baseline reserve appear more vulnerable to functional consequences of structural decline. Early intervention remains critical to preserve brain tissue and cognitive reserve, while next-generation therapies should aim to target CNS-compartmentalized inflammation (e.g., chronic active lesions, microglial activation). Treatment success should thus encompass inflammatory suppression, structural stabilization, cognitive preservation, and prevention of silent progression.

## Limitations

This single-center, real-world observational study has several limitations. The modest sample size, absence of a control group, and non-randomized treatment allocation limit generalizability and preclude causal inference; treatment selection bias may therefore have influenced the characteristics of the cohort [36,37]. In addition, the median follow-up of approximately 19-months captures short-to intermediate-term outcomes but may be insufficient to fully characterize longer-term trajectories.

The structure–cognition associations derived from conditional post-hoc analyses should be interpreted as exploratory and require independent validation. Methodological constraints include reliance on automated volumetry, limited sampling of cognitive assessments and serum NfL, and the absence of complementary biomarkers (e.g., glial fibrillary acidic protein) or advanced imaging modalities such as 7-T MRI, quantitative susceptibility mapping, or positron emission tomography, which may have provided additional mechanistic insights. Furthermore, the use of EDSS as the primary clinical disability measure may underestimate non-motor or patient-reported aspects of disease progression.

With regard to cognition, improvements in test performance should be interpreted cautiously because repeated administration of cognitive measures—particularly the SDMT—may be influenced by practice effects. Previous studies have suggested that a change of approximately 4 points in SDMT performance may represent a clinically meaningful threshold in MS, and similar responder-based thresholds have been preliminarily explored for other BICAMS measures such as the CVLT-II and BVMT-R [38,39]. However, cognitive trajectories in multiple sclerosis are heterogeneous, and modest score increases over time may reflect a combination of preserved performance, measurement variability, and practice effects rather than true cognitive improvement.

Finally, the cognitive battery used in this study (BICAMS) represents a brief screening tool and does not provide the comprehensive neuropsychological assessment offered by more extensive batteries such as minimal assessment of cognitive function in multiple sclerosis- MACFIMS [40]. Consequently, impairments across additional cognitive domains or combined deficits across multiple domains may not have been fully captured. Finally, although cognitive assessments were administered by an experienced neuropsychologist, structured neuropsychological testing may be more difficult to implement in routine clinical practice, which may limit the broader applicability of this approach.

## Conclusions

This real-world longitudinal study suggests that, despite effective peripheral B-cell depletion, ofatumumab-treated RMS patients may experience ongoing but decelerating neurodegeneration. Cognitive performance remained overall stable over time. Such stability may reflect reserve-dependent compensatory mechanisms, with functional decline emerging when these mechanisms are no longer sufficient. These findings highlight the importance of integrating inflammatory, structural, and cognitive dimensions when evaluating treatment response in multiple sclerosis in order to better monitor long-term brain integrity and detect potential silent disease progression.

## Authors contribution

Aurora Zanghì: Drafting/revision of the manuscript for content, including medical writing for content, Study concept or design, Analysis or interpretation of data.

Paola Sofia Di Filippo, Drafting/revision of the manuscript for content, including medical writing for content, Major role in the acquisition of data, Analysis or interpretation of data.

Carlo Robusto, Drafting/revision of the manuscript for content, including medical writing for content, Major role in the acquisition of data.

Maria Claudia Moretti, Drafting/revision of the manuscript for content, including medical writing for content, Major role in the acquisition of data.

Carlo Avolio, Drafting/revision of the manuscript for content, including medical writing for content, Major role in the acquisition of data.

Emanuele D’Amico, Drafting/revision of the manuscript for content, including medical writing for content, Major role in the acquisition of data, Study concept or design, Analysis or interpretation of data, Supervision, Final approval.

Emanuele D’Amico is the guarantor of the manuscript.

## Data availability

Data will be shared upon reasonable request to corresponding author.

## Funding

No funding was received toward this work.

## Declaration of competing interests

Aurora Zanghì, nothing to disclose related to the submitted manuscript.

Paola Sofia Di Filippo, nothing to disclose related to the submitted manuscript.

Carlo Robusto, nothing to disclose related to the submitted manuscript.

Maria Claudia Moretti, nothing to disclose related to the submitted manuscript.

Carlo Avolio, nothing to disclose related to the submitted manuscript.

Emanuele D’Amico, nothing to disclose related to the submitted manuscript.
