# Supplementary material for: Cognitive and structural brain changes in ofatumumab-treated multiple sclerosis: A longitudinal study
Source: Neurotherapeutics. 2026 Jun 3;23(4):e00922. doi: 10.1016/j.neurot.2026.e00922 (PMC13255068; doi:10.1016/j.neurot.2026.e00922)
Supplement: Multimedia component 1 [file mmc1.pdf]

Supplementary Table 1. T1-weighted sequences acquisition parameters.

T1-weighted acquisition										
**Scanner vendor**	**Scanner model**	**Magnetic field**	**TR (ms)**	**TE (ms)**	**Inversion time (ms)**	**Matrix size**	**FOV**	**Resolution (mm)**	**Flip angle**	**Acquisition type**
Siemens	Skyra	3 T	2300	2.3	900	256x256	240 x 240	0.9 x 0.9 0.9	8	3D
Philips	Achieva dStream	1.5 T	7.5	3.4	n.a.	288x288	256x256	0.9 x 0.9 x 0.9	8	3D

FOV, field of view; TE, echo time; TI, inversion time; TR, repetition time

Supplementary Table 2. Longitudinal rates of cortical thickness change during ofatumumab treatment.

		APC ≤12 months (%/year)	APC >12 months (%/year)	Difference in APC	p-value (slope change)
**Temporal lobe**	Enthorinal	-1.19%	+3.03%	+4.22%	0.18
	Temporal pole	+1.10%	+0.47%	-0.63%	0.54
	Superior temporal	-1.73%	+0.19%	+1.92%	0.37
	Inferior temporal	-2.47%	-1.39%	+1.08%	0.56
	Banks superior temporal sulcus	-1.40%	-0.55%	+0.85%	0.67
	Middle temporal	-1.01%	-0.21%	+0.80%	0.68
	Transverse temporal	-1.85%	+1.54%	+3.39%	0.19
**Parietal lobe**	Paracentral	-1.56%	-0.80%	+0.76%	0.72
	Postcentral	-1.90%	-0.02%	+1.88%	0.38
	Precuneus	-2.02%	-0.99%	+1.03%	0.54
	Superior parietal	-2.90%	-1.11%	+1.79%	0.37
	Supra marginal	-2.30%	-0.39%	+1.91%	0.44
	Inferior parietal	-2.47%	-1.39%	+1.08%	0.56
**Occipital lobe**	Pericalcarine	-0.62%	-0.31%	+0.31%	0.88
	Fusiform	-0.43%	0.38%	+0.81%	0.68
	Lateral occipital	-3.20%	-0.56%	-2.64%	0.15
	Cuneus	-1.92%	+0.66%	+2.58%	0.20
	Lingual	-0.64%	+0.43%	-1.07%	0.52
**Limbic lobe**	Rostral anterior cingulate	+0.44%	+0.56%	+0.12%	0.93
	Caudal anterior cingulate	+0.12%	+0.62%	+0.50%	0.76
	Posterior cingulate	+0.40%	+1.50%	+1.10%	0.54
	Parahippocampal	+1.51%	+2.40%	+0.89%	0.74
	Isthmus cingulate	-1.18%	+2.43%	+3.61%	0.12
	Insula	-0.93%	-0.23%	+0.70%	0.62
**Frontal lobe**	Caudal middle frontal	-2.72%	-1.89%	+0.83%	0.63
	Pars triangularis	-2.71%	-1.90%	+0.81%	0.66

	Rostral middle frontal	-2.74%	-1.71%	+1.03%	0.58
	Lateral orbitofrontal	-2.22%	-1.77%	+0.45%	0.80
	Precentral gyrus	-1.89%	-0.02%	+1.87%	0.38
	Pars opercularis	-2.46%	-1.17%	+1.29%	0.50
	Superior frontal	-1.42%	-1.45%	-0.03%	0.98
	Medial orbitofrontal	-0.08%	-1.21%	-1.13%	0.53
	Frontal pole	+0.22%	-2.69%	-2.91%	0.21
	Pars orbitalis	-2.72%	-2.00%	+0.72%	0.74
**Mean CTh**		−1.67%/	−0.62%	+1.05%	0.47

APC: annual percentage change derived from log-transformed mixed-effects models.

*adjusted for sex and CDA at baseline; random intercepts for subject and MRI protocol.

Supplementary Table 3. Fixed-effects estimates from linear mixed-effects models (random intercept for participant) for three cognitive outcomes (SDMT, BVMT-R, and CVLT-II raw scores).

**SDMT**				
**Predictor**	**Beta**	**SE**	**95% CI (lower, upper)**	**p-value**
Intercept	33.435	4.234	25.005, 41.866	**< 0.001**
CDA	-0.401	0.135	-0.669, -0.133	**0.004**
Sex[a]	-0.443	2.173	-4.769, 3.884	0.839
Education	1.205	0.316	0.576, 1.834	**< 0.001**
Time on ofatumumab treatment	3.788	1.228	1.348, 6.229	**0.003**
Post-12-month slope change	0.213	1.594	-2.956, 3.383	0.894
**BVMT-R**				
**Predictor**	**Beta**	**SE**	**95% CI (lower, upper)**	**p-value**
Intercept	13.054	2.675	7.726, 18.382	**< 0.001**
CDA	-0.170	0.085	-0.340, -0.001	**0.049**
Sex[a]	0.897	1.373	-1.838, 3.632	0.516
Education	0.794	0.199	0.397, 1.191	**< 0.001**
Time on ofatumumab treatment	2.111	0.832	0.457, 3.764	**0.013**
Post-12-month slope change	0.222	1.081	-1.927, 2.371	0.838
**CVLT-II**				
**Predictor**	**Beta**	**SE**	**95% CI (lower, upper)**	**p-value**
Intercept	28.367	3.747	20.905, 35.829	**< 0.001**
CDA	-0.334	0.121	-0.574, -0.095	**< 0.001**
Sex[a]	4.542	1.924	0.710, 8.375	**0.021**
Education	1.409	0.279	0.854, 1.964	**< 0.001**
Time on ofatumumab treatment	4.580	1.585	1.432, 7.728	**0.005**
Post-12-month slope change	-0.642	2.068	-4.750, 3.467	0.757

[a], male as a reference;

BVMT-R, Brief Visuospatial Memory Test–Revised; CI, Confidence interval; CVLT-II, California Verbal Learning Test–Second Edition; SDMT, Symbol Digit Modalities Test; SE, Standard error.

*****Values are reported as beta, standard error (SE), 95% confidence interval (CI), and p-value for each predictor.

Each model includes time on ofatumumab treatment as a continuous variable with a piecewise change in slope at 12 months, sex, education and CDA as covariates, with a random intercept for participant. Estimates are reported as β, standard error (SE), 95% confidence interval (CI), and p-value.

Supplementary Table 4. Linear mixed-effects models of SDMT raw score as outcome, testing the association with longitudinal MRI changes in cortical thickness (CTh), thalamic volume (ThalVol), and deep gray matter volume (DGMVol).

**CTh changes → SDMT model**				
**Predictor**	**Beta**	**SE**	**95% CI (lower, upper)**	**p-value**
Intercept	36.222	17.380	1.644, 70.800	**0.040**
CDA	-0.241	0.130	-0.499, 0.016	0.066
Sex[a]	0.288	2.018	-3.730, 4.306	0.887
Education	1.026	0.297	0.436, 1.617	**<0.001**
Time on ofatumumab treatment	3.770	0.559	2.660, 4.880	**<0.001**
Baseline EDSS	-3.330	0.897	-5.115, -1.546	**<0.001**
Within-subject changes in CTh[b]	-0.808	2.887	-6.550, 4.933	0.780
Between-subjects changes in CTh[c]	3.466	6.916	-10.293, 17.226	0.618
**ThalVol changes → SDMT model**				
**Predictor**	**Beta**	**SE**	**95% CI (lower, upper)**	**p-value**
Intercept	-114.999	130.748	-373.497, 143.500	0.381
CDA	-0.241	0.121	-0.499, 0.016	0.066
Sex[a]	2.285	2.237	-2.156, 6.725	0.310
Education	0.933	0.268	0.400, 1.466	**<0.001**
Time on ofatumumab treatment	3.706	0.530	2.653, 4.759	**<0.001**
Baseline EDSS	-2.474	0.810	-4.085, -0.864	**0.003**
eTIV	-9.000	0.750	-30.232, 12.232	0.404
Within-subject changes in ThalVol[b]	-9.515	8.451	-26.318, 7.287	0.263
Between-subjects changes in ThalVol[c]	30.104	7.441	15.339, 44.869	**<0.001**
**DGMVol changes → SDMT model**				
**Predictor**	**Beta**	**SE**	**95% CI (lower, upper)**	**p-value**
Intercept	-192.116	138.407	-466.317, 82.085	0.168
CDA	-0.091	0.120	-0.329, 0.148	0.451
Sex[a]	2.034	2.281	-2.497, 6.565	0.375
Education	0.941	0.265	0.413, 1.469	**<0.001**
Time on ofatumumab treatment	3.718	0.560	2.608, 4.829	**<0.001**

Baseline EDSS	-2.718	0.785	-4.280, -1.156	**<0.001**
eTIV	-14.450	12.530	-39.224, 10.324	0.251
Within-subject changes in DGMVol[b]	-7.557	16.785	-40.827, 25.713	0.653
Between-subjects changes in DGMVol[c]	41.074	10.041	21.149, 60.998	**<0.001**

[a], male as a reference;
[b], computed as each observation's deviation from the subject-specific mean;
[c], computed as the subject-specific mean across available observations.

CDA, Conditional Disease Duration covariate; CI, Confidence interval; CTh, Cortical thickness; DGMVol, Deep gray matter volume; EDSS, expanded disability status scale; eTIV, Estimated total intracranial volume; SDMT, Symbol Digit Modalities Test; SE: Standard error; ThalVol, Thalamic volume.

* Each model includes time on ofatumumab treatment, sex, education, baseline EDSS and CDA as covariates, with a random intercept for participant. MRI predictors are decomposed into within-subject change (deviation from each participant's mean over time) and between-subject difference (participant-specific mean). Estimates are reported as β, standard error (SE), 95% confidence interval (CI), and p-value.

Supplementary Table 5.A. Predictors of 12-month clinical and MRI outcomes in treatment-naïve patients (ANCOVA models). Multivariable ANCOVA models assessing the association between baseline serum neurofilament light chain levels (log-transformed sNfL) and 12-month outcomes, including SDMT raw score, thalamic volume, DGM volume, and mean CTh.

**12-months DGM volume**				
**Predictor**	**Beta**	**SE**	**95% CI (lower, upper)**	**p-value**
Intercept	2.414	1.754	-1.258, 6.085	0.185
Baseline DGM volume	1.065	0.143	0.765, 1.365	**<0.001**
Baseline sNfL	0.001	0.015	-0.031, 0.033	0.951
Baseline eTIV	-0.216	0.174	-0.580, 0.148	0.229
Sex[a]	-0.062	0.025	-0.115, -0.009	**0.025**
CDA	0.006	0.003	0.000, 0.012	**0.045**
**12-months mean CTh**				
**Predictor**	**Beta**	**SE**	**95% CI (lower, upper)**	**p-value**
Intercept	2.195	0.585	0.975, 3.415	**0.001**
Baseline mean CTh	0.045	0.232	-0.439, 0.530	0.847
Baseline sNfL	0.057	0.046	-0.039, 0.153	0.232
Sex[a]	0.056	0.061	-0.071, 0.182	0.370
CDA	0.014	0.008	-0.004, 0.031	0.113
**12-months SDMT raw score**				
**Predictor**	**Beta**	**SE**	**95% CI (lower, upper)**	**p-value**
Intercept	10.023	9.182	-9.196, 29.242	0.289
Baseline SDMT raw score	0.772	0.129	0.502, 1.041	**<0.001**
Baseline sNfL	0.732	1.806	-3.048, 4.513	0.690
Sex[a]	0.580	2.287	-4.206, 5.366	0.802
Education	0.206	0.378	-0.585, 0.997	0.592

CDA	-0.174	0.304	-0.811, 0.463	0.574
**12-months ThalVol**				
**Predictor**	**Beta**	**SE**	**95% CI (lower, upper)**	**p-value**
Intercept	2.731	2.879	-3.294, 8.756	0.355
Baseline ThalVol	0.822	0.188	0.430, 1.215	**<0.001**
Baseline sNfL	-0.038	0.025	-0.091, 0.015	0.154
Baseline eTIV	-0.061	0.245	-0.573, 0.451	0.806
Sex[a]	-0.100	0.042	-0.188, -0.013	**0.026**
CDA	0.007	0.005	-0.003, 0.017	0.157

[a], male as a reference;

CDA, Conditional Disease Duration covariate; CI, Confidence interval; CTh, Cortical thickness; DGMVol, Deep gray matter volume; eTIV, Estimated total intracranial volume; SDMT, Symbol Digit Modalities Test; SE: Standard error; sNfL, serum neurofilament light chains; ThalVol, Thalamic volume.

*All models were adjusted for the baseline value of the corresponding outcome and relevant covariates as specified. Regression coefficients (β), standard errors (SE), 95% confidence intervals (CI), and p-values are reported. Volumetric outcomes were log-transformed and adjusted for intracranial volume (log-eTIV).

Supplementary Table 5.B. Predictors of 12-month clinical and MRI outcomes in treatment-naïve patients (ANCOVA models). Multivariable ANCOVA models assessing the association between baseline cerebrospinal fluid neurofilament light chain levels (log-transformed cNfL) and 12-month outcomes, including SDMT raw score, thalamic volume, DGM volume, and mean CTh.

**12-months DGM volume**				
**Predictor**	**Beta**	**SE**	**95% CI (lower, upper)**	**p-value**
Intercept	2.480	1.752	-1.187, 6.148	0.173
Baseline DGM volume	1.076	0.145	0.772, 1.38	<0.001
Baseline cNfL	-0.004	0.009	-0.023, 0.016	0.705
Baseline eTIV	-0.227	0.176	-0.595, 0.141	0.211
Sex[a]	-0.061	0.025	-0.114, -0.008	0.026
CDA	0.006	0.003	0.001, 0.012	0.036
**12-months mean CTh**				
**Predictor**	**Beta**	**SE**	**95% CI (lower, upper)**	**p-value**
Intercept	2.399	0.590	1.17, 3.629	<0.001
Baseline mean CTh	-0.090	0.255	-0.622, 0.441	0.727
Baseline cNfL	0.047	0.030	-0.015, 0.109	0.131
Sex[a]	0.045	0.060	-0.08, 0.17	0.459
CDA	0.016	0.008	-0.001, 0.032	0.056
**12-months SDMT raw score**				
**Predictor**	**Beta**	**SE**	**95% CI (lower, upper)**	**p-value**
Intercept	-0.960	10.337	-22.596, 20.676	0.927
Baseline SDMT raw score	0.857	0.130	0.586, 1.128	<0.001

Baseline cNfL	1.791	1.086	-0.483, 4.064	0.116
Sex[a]	0.078	2.171	-4.465, 4.621	0.972
Education	0.009	0.376	-0.778, 0.796	0.981
CDA	-0.149	0.281	-0.737, 0.44	0.603
**12-months ThalVol**				
**Predictor**	**Beta**	**SE**	**95% CI (lower, upper)**	**p-value**
Intercept	3.538	2.885	-2.501, 9.576	0.235
Baseline ThalVol	0.863	0.191	0.464, 1.262	<0.001
Baseline cNfL	-0.022	0.015	-0.054, 0.009	0.157
Baseline eTIV	-0.142	0.247	-0.659, 0.375	0.573
Sex[a]	-0.102	0.042	-0.19, -0.015	0.024
CDA	0.006	0.005	-0.004, 0.015	0.235

[a], male as a reference;

CDA, Conditional Disease Duration covariate; CI, Confidence interval; cNfL, cerebrospinal fluid neurofilament light chains; CTh, Cortical thickness; DGMVol, Deep gray matter volume; eTIV, Estimated total intracranial volume; SDMT, Symbol Digit Modalities Test; SE: Standard error; ThalVol, Thalamic volume.

* All models were adjusted for the baseline value of the corresponding outcome and relevant covariates as specified. Regression coefficients (β), standard errors (SE), 95% confidence intervals (CI), and p-values are reported. Volumetric outcomes were log-transformed and adjusted for intracranial volume (log-eTIV).

Supplementary Table 6.A. Sensitivity analyses of ANCOVA models* evaluating the association between baseline sNfL levels and 12-month outcomes.

**12-months DGM volume**			
**Predictor**	**Primary ANCOVA model (beta; p-value)**	**ANCOVA excluding IQR/Cook's distance outliers (beta; p-value)**	**ANCOVA with 95th-percentile winsorization (beta; p-value)**
Intercept	2.414; 0.185	4.213; 0.091	2.426; 0.183
Baseline DGM volume	**1.065; <0.001**	**0.887; <0.001**	**1.065; <0.001**
Baseline sNfL	0.001; 0.951	0.045; 0.090	0.002; 0.891
Baseline eTIV	-0.216; 0.229	-0.215; 0.305	-0.217; 0.227
Sex[a]	**-0.062; 0.025**	**-0.074; 0.024**	**-0.062; 0.024**
CDA	**0.006; 0.045**	0.003; 0.478	**0.006; 0.047**
**12-months mean CTh**			
**Predictor**	**Primary ANCOVA model (beta; p-value)**	**ANCOVA excluding IQR/Cook's distance outliers (beta; p-value)**	**ANCOVA with 95th-percentile winsorization (beta; p-value)**
Intercept	**2.195; <0.001**	**2.515; <0.001**	**2.193; <0.001**
Baseline mean CTh	0.045; 0.847	-0.052; 0.822	0.042; 0.857
Baseline sNfL	0.057; 0.232	0.057; 0.361	0.061; 0.216
Sex[a]	0.056; 0.370	-0.021; 0.748	0.056; 0.363
CDA	0.014; 0.113	**0.021; 0.043**	0.013; 0.116
**12-months SDMT raw score**			
**Predictor**	**Primary ANCOVA model (beta; p-value)**	**ANCOVA excluding IQR/Cook's distance outliers (beta; p-value)**	**ANCOVA with 95th-percentile winsorization (beta; p-value)**
Intercept	10.023; 0.289	13.673; 0.186	10.297; 0.283
Baseline SDMT raw score	**0.772; <0.001**	**0.887; <0.001**	**0.769; <0.001**
Baseline sNfL	0.732; 0.690	-2.468; 0.328	0.658; 0.729
Sex[a]	0.580; 0.802	-1.120; 0.689	0.596; 0.797
Education	0.206; 0.592	-0.007; 0.986	0.208; 0.589

CDA	-0.174; 0.574	-0.133; 0.738	-0.172; 0.579
**12-months ThalVol**			
**Predictor**	**Primary ANCOVA model (beta; p-value)**	**ANCOVA excluding IQR/Cook's distance outliers (beta; p-value)**	**ANCOVA with 95th-percentile winsorization (beta; p-value)**
Intercept	2.731; 0.355	3.956; 0.366	2.736; 0.357
Baseline ThalVol	**0.822; <0.001**	**0.808; 0.005**	**0.819; <0.001**
Baseline sNfL	-0.038; 0.154	0.022; 0.624	-0.037; 0.179
Baseline eTIV	-0.061; 0.806	-0.149; 0.641	-0.059; 0.813
Sex[a]	**-0.100; 0.026**	-0.086; 0.131	**-0.101; 0.026**
CDA	0.007; 0.157	0.002; 0.775	0.007; 0.162

[a], male as a reference;

CDA, Conditional Disease Duration covariate; CI, Confidence interval; CTh, Cortical thickness; DGMVol, Deep gray matter volume; eTIV, Estimated total intracranial volume; IQR, interquartile ranges; SDMT, Symbol Digit Modalities Test; SE: Standard error; sNfL, serum neurofilament light chains; ThalVol, Thalamic volume.

* For each outcome (DGM volume, mean CTh, SDMT raw score, and thalamic volume), three ANCOVA models are reported: the primary model, a model excluding subjects identified as outliers based on the interquartile range (IQR) and Cook's distance, and a model with baseline sNfL values winsorized at the 95th percentile. All models were adjusted for the corresponding baseline outcome value and relevant covariates, as indicated. Regression coefficients (beta) and associated p-values are shown.

Supplementary Table 6.B. Sensitivity analyses of ANCOVA models evaluating the association between baseline cNfL levels and 12-month outcomes.

**12-months DGM volume**			
**Predictor**	**Primary ANCOVA model (beta; p-value)**	**ANCOVA excluding IQR/Cook's distance outliers (beta; p-value)**	**ANCOVA with 95th-percentile winsorization (beta; p-value)**
Intercept	2.48; 0.173	2.744; 0.285	2.416; 0.186
Baseline DGM volume	**1.076; <0.001**	**0.977; <0.001**	**1.067; <0.001**
Baseline cNfL	-0.004; 0.705	0.002; 0.889	-0.001; 0.953
Baseline eTIV	-0.227; 0.211	-0.173; 0.469	-0.217; 0.231
Sex[a]	**-0.061; 0.026**	**-0.072; 0.048**	**-0.062; 0.025**
CDA	**0.006; 0.036**	**0.007; 0.052**	**0.006; 0.039**
**12-months mean CTh**			
**Predictor**	**Primary ANCOVA model (beta; p-value)**	**ANCOVA excluding IQR/Cook's distance outliers (beta; p-value)**	**ANCOVA with 95th-percentile winsorization (beta; p-value)**
Intercept	**2.399; <0.001**	**2.645; <0.001**	**2.439; <0.001**
Baseline mean CTh	-0.090; 0.727	-0.135; 0.584	-0.140; 0.590
Baseline cNfL	0.047; 0.131	0.036; 0.345	0.061; 0.081
Sex[a]	0.045; 0.459	-0.03; 0.640	0.048; 0.425
CDA	0.016; 0.056	**0.022; 0.014**	0.016; 0.053
**12-months SDMT raw score**			
**Predictor**	**Primary ANCOVA model (beta; p-value)**	**ANCOVA excluding IQR/Cook's distance outliers (beta; p-value)**	**ANCOVA with 95th-percentile winsorization (beta; p-value)**
Intercept	-0.960; 0.927	3.370; 0.801	-0.729; 0.949
Baseline SDMT raw score	**0.857; <0.001**	**0.919; <0.001**	**0.848; <0.001**
Baseline cNfL	1.791; 0.116	0.483; 0.757	1.798; 0.163
Sex[a]	0.078; 0.972	-0.528; 0.846	0.273; 0.902
Education	0.009; 0.981	-0.041; 0.919	0.021; 0.957

CDA	-0.149; 0.603	-0.231; 0.481	-0.156; 0.591
**12-months ThalVol**			
**Predictor**	**Primary ANCOVA model (beta; p-value)**	**ANCOVA excluding IQR/Cook's distance outliers (beta; p-value)**	**ANCOVA with 95th-percentile winsorization (beta; p-value)**
Intercept	3.538; 0.235	3.471; 0.403	3.411; 0.266
Baseline ThalVol	**0.863; <0.001**	**0.843; 0.003**	**0.839; <0.001**
Baseline cNfL	-0.022; 0.157	-0.006; 0.789	-0.017; 0.348
Baseline eTIV	-0.142; 0.573	-0.131; 0.675	-0.119; 0.644
Sex[a]	**-0.102; 0.024**	-0.088; 0.125	**-0.105; 0.024**
CDA	0.006; 0.235	0.005; 0.362	0.006; 0.255

[a], male as a reference;

CDA, Conditional Disease Duration covariate; CI, Confidence interval; cNfL, cerebrospinal fluid neurofilament light chains; CTh, Cortical thickness; DGMVol, Deep gray matter volume; eTIV, Estimated total intracranial volume; IQR, interquartile ranges; SDMT, Symbol Digit Modalities Test; SE: Standard error; ThalVol, Thalamic volume.

*For each outcome (DGM volume, mean CTh, SDMT raw score, and thalamic volume), three ANCOVA models are reported: the primary model, a model excluding subjects identified as outliers based on the interquartile range (IQR) and Cook's distance, and a model with baseline sNfL values winsorized at the 95th percentile. All models were adjusted for the corresponding baseline outcome value and relevant covariates, as indicated. Regression coefficients (beta) and associated p-values are shown.
